# Harris' Principles and Practice of Dentistry

**Published:** 1889-04

**Authors:** 


### Bibliographical.

---

HARRIS' PRINCIPLES AND PRACTICE OF DENTISTRY.—*Twelfth Edition. Revised and Edited by Ferdinand J. S. Gorgas, A. M., M. D., D. D. S., 1889.*—This twelfth edition of our Standard Harris is as large as a city directory. It contains 1086 illustrations, and 1222 pages, including a complete index. It comes from the well-known press of P. Blakiston, Son & Co., of Philadelphia, and we need not add the workmanship, in the way of printing, binding, etc., is in keeping with the character of this well-known house.

What would "American Dentistry" be to-day if there never had been a "Harris' Principles and Practice?"

Where is there a dentist in this country, now practicing, who has not poured over the pages of Harris?

How well we remember the early edition, which was handed to us some thirty years ago by our dear old preceptor, on the day upon which we entered his office as a student of dentistry. What a wonderful book it seemed to us then, with its anatomy, pathology, therapeutics, surgery and mechanics.

It *was* a wonderful book, and this new edition of 1889 *is* a wonderful book.

The complete picture of the *science* and *art* of dentistry was displayed in the old edition, and just as fully in the new edition, and it has taken some seven hundred more pages to present the picture of to-day.

Professor Gorgas has proved himself eminently fitted for this work, and seems not to have omitted a single point in any department of the science or art of this active specialty of medicine, recognized by the profession this year in this edition. It seems to be really a complete exposition of dentistry. We find, for instance, in the discussion of the causes of dental caries, the late experiments of Dr. W. D. Miller, of Berlin, and the doctrines of Dr. Geo. Watt. In Pathology, we find references to the work of Dr. G. V. Black. In orthodontia, all the favorite methods of our most prominent operators are illustrated.

In "Operative Dentistry," the beginner can get from this edition all that it is possible to obtain from description of methods, and the old operator can get a review of the methods of famous operators. Crown and Bridge Work is as fully illustrated in this volume as it has been in any work specially devoted to this one subject. In "Mechanics, or Dental Prosthesis," everything that we can think of, from how to make a spiral spring, to the method of constructing an aluminum cast plate, and from a bench vise to a Knapp nitrous oxide combination blow-pipe, seems to be fully represented by descriptions and illustrations. It is a wonderful book! A whole dental library in itself! An indispensable book! A complete system of "dentistry!" It is a "Webster's Unabridged," and should lie within handy reach of every dentist and every dental student in the world.—*Cincinnati Medical Journal.* C. M. W.

